# Analysis of Combinatorial Regulation: Scaling of Partnerships between Regulators with the Number of Governed Targets

**DOI:** 10.1371/journal.pcbi.1000755

**Published:** 2010-05-27

**Authors:** Nitin Bhardwaj, Matthew B. Carson, Alexej Abyzov, Koon-Kiu Yan, Hui Lu, Mark B. Gerstein

**Affiliations:** 1 Program in Computational Biology and Bioinformatics, Yale University, New Haven, Connecticut, United States of America; 2 Bioinformatics Program, University of Illinois at Chicago, Chicago, Illinois, United States of America; 3 Department of Molecular Biophysics and Biochemistry, Yale University, New Haven, Connecticut, United States of America; 4 Department of Computer Science, Yale University, New Haven, Connecticut, United States of America; University of Chicago, United States of America

## Abstract

Through combinatorial regulation, regulators partner with each other to control common targets and this allows a small number of regulators to govern many targets. One interesting question is that given this combinatorial regulation, how does the number of regulators scale with the number of targets? Here, we address this question by building and analyzing co-regulation (co-transcription and co-phosphorylation) networks that describe partnerships between regulators controlling common genes. We carry out analyses across five diverse species: *Escherichia coli* to human. These reveal many properties of partnership networks, such as the absence of a classical power-law degree distribution despite the existence of nodes with many partners. We also find that the number of co-regulatory partnerships follows an exponential saturation curve in relation to the number of targets. (For *E. coli* and *Bacillus subtilis*, only the beginning linear part of this curve is evident due to arrangement of genes into operons.) To gain intuition into the saturation process, we relate the biological regulation to more commonplace social contexts where a small number of individuals can form an intricate web of connections on the internet. Indeed, we find that the size of partnership networks saturates even as the complexity of their output increases. We also present a variety of models to account for the saturation phenomenon. In particular, we develop a simple analytical model to show how new partnerships are acquired with an increasing number of target genes; with certain assumptions, it reproduces the observed saturation. Then, we build a more general simulation of network growth and find agreement with a wide range of real networks. Finally, we perform various down-sampling calculations on the observed data to illustrate the robustness of our conclusions.

## Author Summary

A regulatory network consists of regulators such as transcription factors or kinases that control the expression or activity of their target genes. Almost always, there are multiple regulators partnering together to control their targets. Compared to more commonplace contexts, these regulators can be thought of as managers in a social or corporate setting controlling their common subordinates. One interesting question that we address here in this study is how the number of governing regulators scales with the number of governed targets. We build and analyze co-regulation (co-transcription and co-phosphorylation) networks that describe partnerships between regulators controlling common genes. We use a simple framework across five species that demonstrate a wide range of evolution: *Escherichia coli* to human. The analysis reveals many properties of partnership networks and shows that the number of co-regulatory partnerships follows an exponential saturation curve with the number of targets. To gain more intuition, we explore more commonplace contexts and find that exponential saturation relationship also exists in several social networks. Finally, we propose a simple model to explain this relationship that also exists in a simulated evolutionary environment.

## Introduction

Regulating the spatial and temporal activity of genes is essential to the smooth functioning of biological processes in the cell. The two primary processes for mediating this regulation are transcription and phosphorylation. As a part of the former type of regulation, certain proteins called transcription factors (TFs) bind to specific places in the genome and regulate the expression of target genes (TGs). Similarly, under phosphorylation, a specific set of proteins (collectively called kinases) add phosphate groups to certain amino acids, thus regulating the activity of the protein in a post-translational manner. These sets of regulatory interactions can be represented as a directed graph with edges directing from regulators to target genes [1], [2], [3]. Many previous studies have focused on the topological properties of molecular networks and have uncovered some design principles, such as the scale-free topology [4], [5], modularity [6], [7], [8], disassortativeness [9], and enrichment in certain network motifs [10], [11], [12], [13]. Many of these properties, in addition to others, are thought to promote robustness [4], [9], [14], [15], [16].

The regulators (both TFs and kinases) perform their function mostly in combination with other regulators under different spatial and/or temporal conditions. This is referred to as combinatorial regulation and allows for a sophisticated response to multiple conditions in the environment, integration of multiple signaling inputs, and generation of highly specific outputs with the help of a relatively small number of regulators. Many structural and biochemical studies have revealed several key features of the co-regulatory partnerships between different TFs such as modular organization of different kinds of hubs [17] and existence of a distributed architecture behind the scale-free transcriptional regulatory network [18]. There has been progress towards finding and reconstructing aspects of the cellular program of combinatorial transcriptional control [19], [20], [21], [22], [23], their integration with diverse data [24], [25] and their robustness to rewiring [26]. The genome-scale principles of the partnerships between transcription factors, however, remain largely unexplored, with the exception of a few earlier studies which focused on certain aspects of these principles towards different aims such as the design of in-silico transcriptional logic gates using an evolutionary algorithm [27] and the integration of metabolic and transcriptional regulatory networks [28].

Such partnerships to manage common subordinates are also readily seen in many commonplace social contexts. For example, in an academic institution (say a high school), there are multiple teachers supervising the same set of students and hence they have partnership interactions amongst themselves. One interesting question in this regard is, both in commonplace social settings and in molecular networks, how the size of the governing body scales with that of the governed population.

To address this question, we generate partnership networks from transcriptional networks for five species spanning a large evolutionary period and a phosphorylation network for yeast. To bolster our observations, we also perform the same analysis for human modification network that includes many other kinds of post-translational modifications such as acetylation, carboxylation and nitration (included in the supplementary text). These networks, which we call ‘partnership’ networks, describe pairings between regulators to regulate common targets. We analyze both regulatory and co-regulatory connectivity of different regulators and reveal an exponential saturation relationship between the number of partners and the number of targets. This relationship indicates that the number of partners increases exponentially with the number of targets but eventually saturates, indicating that only a limited number of partners are required to regulate an increasing number of targets. Mapping of similar behavior in social settings provides some intuition about the regulatory apparatus active in the cell. To this end, we analyze some directed social networks and find that they exhibit an exponential saturation relationship between the number of ‘supervisors’ and their output. A simple model that explains this relationship and fits the framework is also presented.

## Results

**Figure 1:**
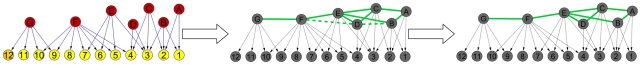
Obtaining a co-regulation network from a regulatory network. We first placed an edge between two TFs (or kinases) if they co-regulated (or co-phosphorylated) at least one common target gene. 1,000 random networks of the same degree distribution were then generated. A co-regulation coefficient (CC) for each pair of regulators was defined as the ratio of the average number of genes co-regulated in real network versus random networks. Only those edges with CC>1 were retained (solid green lines in the last network). In this paper, we study the scaling of partners of each regulator (green edges) with the number of targets (outgoing gray edges).

Five evolutionarily diverse species were chosen for the analysis (*E. coli*, yeast, mouse, rat and human) as transcriptional regulatory data is most plentiful for them (Table 1, see Materials and Methods). However, the phosphorylation network was analyzed only for yeast as the data for other species is very sparse (Table 1). Beginning with the regulatory network, we built the co-regulatory network by first placing an edge between two regulators (TFs or kinases) if they regulate the same target gene (figure 1). By comparison to 1,000 control networks of the same degree distribution as the original network, only those co-regulatory associations that were more frequent than random ones were kept (see Materials and Methods).

**Table 1:** The sizes of the regulatory networks (transcription and phosphorylation) for each species.

Network type	Species	Number of regulators	Number of targets	Number of interactions
Transcription	*E. coli*	160	1,420	3,123
Transcription	Yeast	157	4,410	12,873
Transcription	Mouse	144	1,092	2,403
Transcription	Rat	91	461	1,092
Transcription	Human	156	3,032	6,896
Phosphorylation	Yeast	87	1,337	4,083
Modification	Human	518	1,218	2,782

### Connectivity of the partnership network

**Figure 2:**
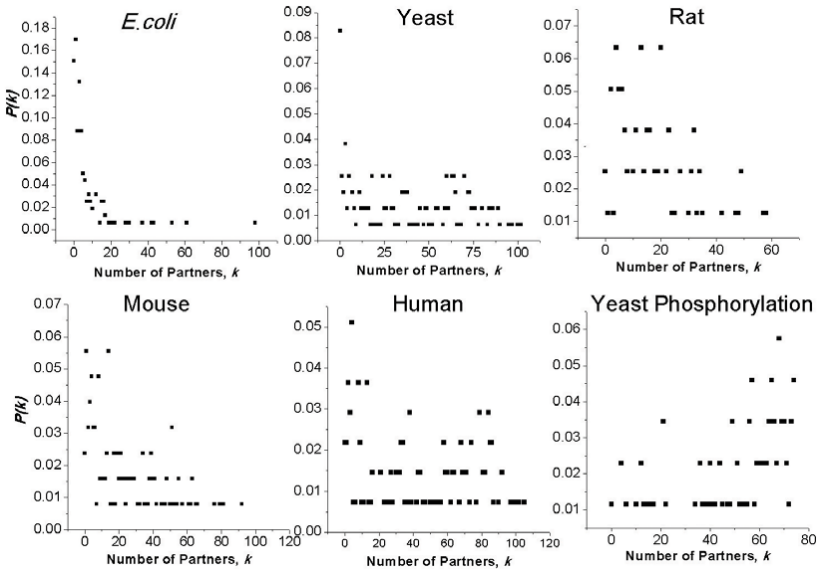
Connectivity of the partnership networks in several organisms. (a–e) The transcription network of five species, (f) the phosphorylation network in yeast. With the exception of *E. coli*, which shows inhomogeneous connectivity (only a few regulators with a large number of partners and large number of regulators with a few partners), all other species display homogenous connectivity.

Previous studies have shown that regulatory networks show inhomogeneous connectivity [4], [5], [9] where very few proteins have a disproportionately high number of links and a large number of proteins have very few links. Under an inhomogeneous architecture, the connectivity distribution *P(k)* falls exponentially with the connectivity, *k*, i.e., *p(k)*∼*k^−γ^* for some *γ*>0. We find that co-regulation networks (both co-transcription and co-phosphorylation), on the other hand, display homogeneous connectivity (apart from *E. coli*, further discussed below) i.e., *P(k)* is rather evenly distributed across different values of *k* (the number of partners, Figure 2). Although, rat and mouse display a negative correlation between *P(k)* and *k*, the relationship does not follow a power-law (*R^2^* = 0.07 and 0.3 for rat and mouse, respectively). Earlier, a similar distributed architecture has been reported for yeast [18]. While such architecture makes the network more sensitive to random removal of a large fraction of nodes, it increases the robustness against targeted attacks on highly connected nodes. The absence of a power-law-like distribution also suggests that there are no hubs in the partnership network. This means that there is no single regulator (or very few regulators) that most regulators partner with, rather there is a uniform distribution of partnerships among regulators.

### Connectivity of regulation vs. co-regulation networks

**Figure 3:**
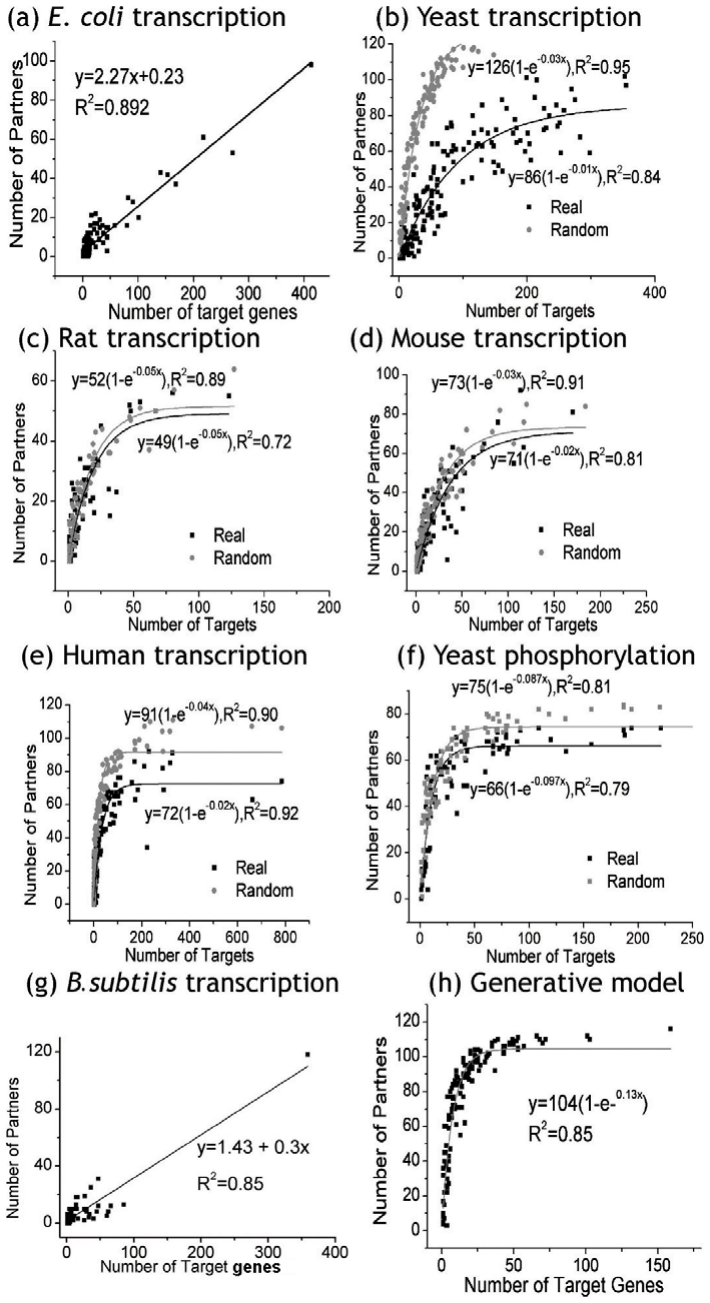
The number of partners vs. the number of target genes for each regulator. (a–e) The transcription network of five species, (f) the phosphorylation network in yeast, (g) the transcription network of *B. subtilis* and (h) the generative model. Black and gray lines correspond to real and random networks respectively. Random networks were generated by shuffling the edges in real networks while maintaining the in- and out-degree of each node. The best fit line and corresponding R^2^ value is indicated for each sub-graph.

To investigate the relationship between regulatory and co-regulatory interactions, we plotted the number of targets for each regulator (the connectivity in the regulatory network) vs. the number of its partners (the connectivity in the co-regulatory network, Figure 3). We find that for *E. coli*, the number of co-regulatory partners increases linearly with the number of target genes. The relationship was retained when the outliers, those proteins with a high number of partners and targets, were excluded from the analysis. To investigate whether this behavior is found in other bacteria as well, we examined *B. subtilis* and found that the same relationship holds (Figure 3g), suggesting that this might be a general feature of the bacterial kingdom.

Notably, this relationship is different in other species for which the number of partners initially increases exponentially with the number of targets but saturates at a certain value for large numbers of targets. In addition to phosphorylation network in yeast, the same relationship is also found in modification network for human (Figure 1 in text S1 and Materials and Methods for details). This relationship can be fitted with the exponential saturation curve, *f(x) = a(1−e^−bx^)*, where *a* and *b* are non-negative numbers. *a* equals the saturation limit of *f(x)* and *b* determines how quickly *f(x)* approaches *a*. Interestingly, for all four species, the limiting number of partners, *a*, equals roughly half the total number of potential regulators, meaning that these regulators only partner with at most half the number of partners available in the network.

### Different behavior in bacteria

**Figure 4:**
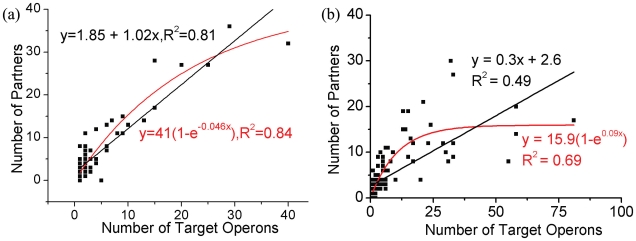
The number of partners vs. the number of target operons. (a) *E. coli* and (b) *B. subtilis*. The exponential saturation curve (in red) shows a slightly better fit than the linear curve (in black) for both species.

As shown above, *E. coli*, along with another bacterium, *B. subtilis*, demonstrates a linear relationship between the number of targets and the number of partners, unlike other species that display an exponential saturation relationship. However, we believe that there is indeed no anomaly; a linear relationship is seen because the saturation tail of the relationship is not reached due to insufficient coverage or sampling so only the beginning of the exponential curve is seen (which is nearly linear). In other words, number of partners does not reach its saturation limit (the tail of the exponential curve) so only the beginning linear part is manifested. We further reason that this is due to the arrangement of several genes into operons which are regulated by the same promoter region in bacteria. Arrangement into operons reduces the ‘effective’ number of distinct genes available. More specifically, in the context of the exponential saturation equation, for smaller *x* (target genes), *e^−bx^* roughly equals *−bx* and *a(1−e^−bx^)* approximates to *a(1+bx)* hence giving a linear equation in *x* which is what we observe. If the difference is indeed due to the presence of operons, one would see the same relationship in other bacteria species if genes, with recalibration, were grouped together by operons. Indeed, we observe that the lagging tail part of the exponential saturation relationship between the number of partners and the number of operons shows up (Figure 4) for both *E. coli* and *B. subtilis*. The same observation is obtained when points on the upper right corner of the plot are removed for *E. coli* for which the relationship seems a little weaker in Figure 4a (Figure 2 in text S1). This indicates that only the linear behavior is manifested in the case of *E. coli* due to arrangement of genes into operons as the tail part of the exponential part is not reached.

### Comparisons to social networks

**Figure 5:**
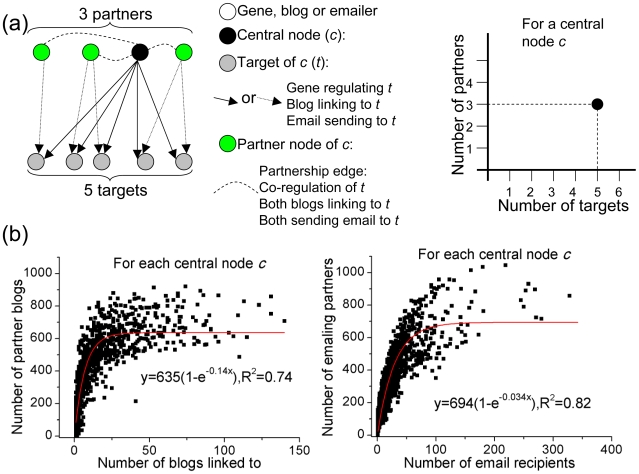
A comparison using directed social networks. (a) A schematic of the process of analyzing the number of targets and partners for a node of interest (black node, labeled *c*). It can be a gene and its targets are the genes it regulates and its partners are other regulators that control at least one common target. In social contexts studied here, a node can also be a blog with other blogs that it links to as its targets, and the other blogs that link to same target blogs as its partners. Similarly, it can also be an email user whose targets are the users he/she sends an email to and her/his partners are other users that email at least one common user. We study the scaling of partners (y-axis) with the number of targets (x-axis). (b) The number of blogs a user links his/her blogs to (x-axis) vs. the number of blogs which point links to the same blogs (y-axis). Each data point corresponds to a blog in the blogs network (Left panel). The number of recipients a user sends an email to vs. the number of other users who email the same recipients (Right Panel). Each data point corresponds to a user (who sends an email) in the email network.

The World Wide Web creates an infinitely rich network between users with various kinds of interactions: exchange of emails, friendships on social networking sites, commenting on blogs and on photo-sharing sites like flickr and other interactions (such as rating videos and becoming a fan) on YouTube. Some of these are directed networks provide easy templates for comparisons to biological networks. To gain more intuition into the saturation phenomenon, we examined two directed social networks for the same relationship. We studied a blog linkage network that consisted of inter-linked blog entries where blogs are nodes, links to them are edges between them and a ‘co-link’ occurs when two blogs link to a common blog (Figure 5a). We also studied an email network obtained using a set of emails exchanged amongst users that share a ‘co-send’ partnership if they send an email to a common user. We found that both these networks displayed the same kind of exponential saturation relationship between the output (the number of out-going links or email recipients) and the number of partners (co-linkers or co-senders) (Figure 5b). This suggests that in social networks as well, the size of partnership network saturates at a certain value even as the output of the group gets exponentially complex, highlighting the similarities between the organizational structure of social and biological networks.

A limit on the size of the social network an individual can develop has been reported previously as well. It has been suggested before that a human brain allows a stable network of about 150 (known as the ‘Dunbar number’) [29]. Similarly, the average number of “friends” on social networking sites like Facebook has been observed to be 120 [30]. These observations and our results above are indirectly related: setting a cap on the number of individuals one interacts with loosely limits the number of other individuals (the partners) that interact with the same group.

### Comparisons between real and random networks

We performed various comparisons between random and real networks, and present two models to describe this process: we build a simple theoretical model that reproduces the real networks with certain assumptions and for a range of parameters and then follow with a more general simulation of network growth to match in a wider range.

First, we investigated randomized networks of the same topology by generating control networks, maintaining the same in- and out-degree of each node in the model organism networks. In each case, the saturation limit for real networks was lower than that for random networks (Figure 3b–f), indicating that fewer pairings between regulators are possible in real scenarios than random. This might be due to the fact that in real networks all regulators have specific co-targets and thus partner only with certain other regulators. For example, most of the regulators are active only in specific tissues and thus can only partner with other regulators that are active in the same tissues. Another plausible reason for this might be that certain co-regulators are more likely to partner with each other; for instance, several TF complexes are formed by proteins of specific structural classes, such as homeo-domains or bZIPs. Similarly, the finite length of the regulatory region of the DNA might also explain a lower limit in real transcriptional networks – binding of a protein physically occludes other regulatory sites on the DNA and thus limits the number of partners regulating the same DNA. This highlights the specificity of regulatory interactions in the cell.

### A simple theoretical model to describe the saturation process

**Figure 6:**
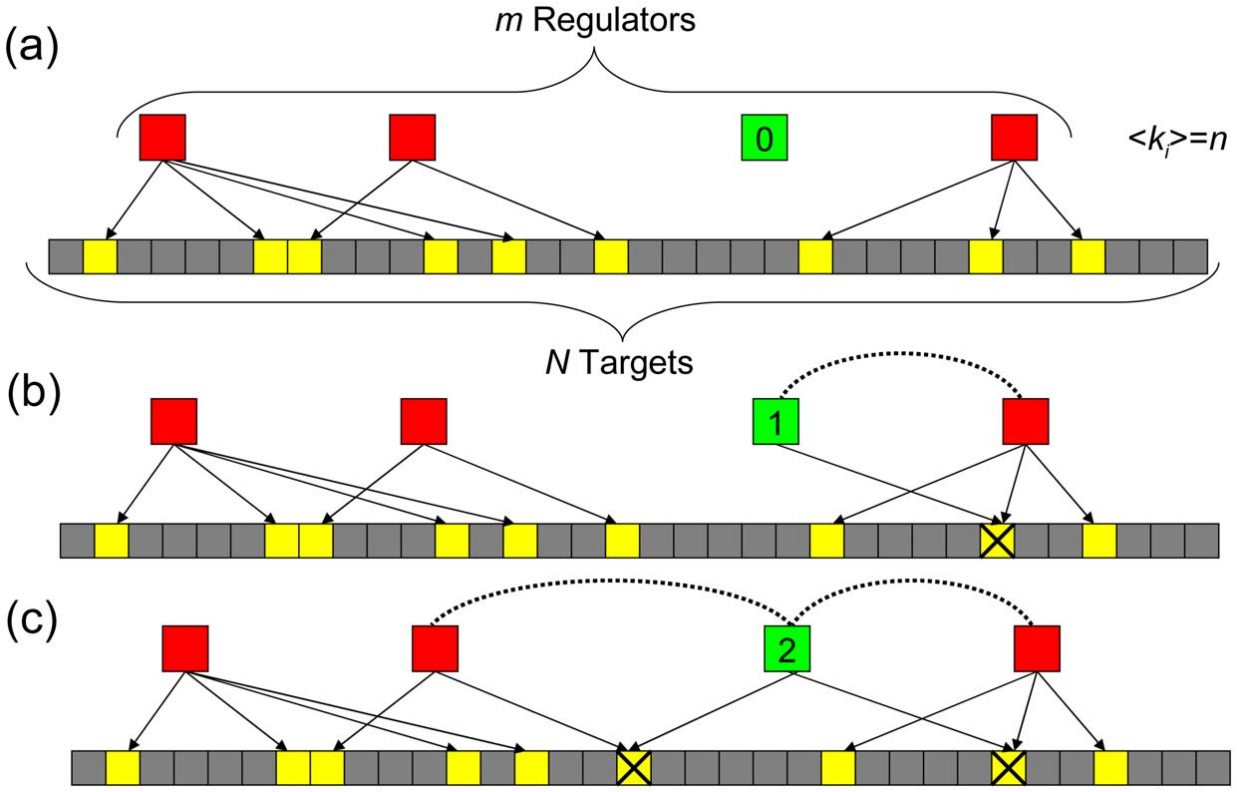
A model describing the growth of co-regulation networks. (a) Initially, there are no co-regulatory interactions between regulators. (b) Upon growth, the new regulator must (shown in green) begin regulating an already regulated gene (the yellow box marked with a cross) in order to gain a co-regulation partnership (dashed line). (c) In order to gain another partnership, a regulator must regulate genes regulated by other regulators (the left yellow box marked with a cross) besides its partners. The number in each box represents the current number of targets for that regulator.

Now, we present a simple model that describes the growth of co-regulation partnerships with certain assumptions resulting in an exponential saturation relationship. For simplicity, we consider a total of *m* regulators and *N* available targets. On average, each regulator has *n* targets making a total of *nm* regulated targets (Figure 6a). For a specific regulator, *i*, the number of targets is *k_i_*, so <*k_i_*> = *n* averaged over all *i*, *i* = 1 to *m*. We assume that the pool of targets is large, resulting in the number of genes regulated by two or more regulators being small. We further assume that during the course of evolution, regulators acquire target genes randomly. Let *f_i_* be the number of partners for the regulator *i*. In the subsequent discussion, although we talk about a specific regulator (*i*) acquiring partners, we drop the subscript. Now, for a regulator with no partners the expected increase of co-regulatory partners acquired, 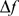, upon adding a new gene, 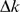, equals the fraction of targets that are already being regulated, i.e., 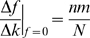 (Figure 6b). For regulators with one partner, a co-regulatory partner will be acquired only if the new gene it targets is not yet regulated by its existing co-regulatory partners (there are *(m−1)n* of them), i.e. .
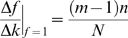
. Recall that we assume that only a few genes are regulated by multiple regulators hence we can neglect any co-regulation between regulators. Continuing in the same way, the expected number of co-regulatory partners acquired given it already has *f* partners is 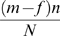. Therefore, the rate of increase of new co-regulation partners with respect to the number of targets when averaged over many genes becomes 
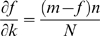
 where *k* is the number of target genes. Solving this differential equation gives the solution 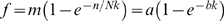, where *a* = *m* (the limiting number of partners) and *b* = *n/N* (the fraction of total genes regulated by each regulator on an average). This equation represents the exponential saturation relationship observed above for all networks. Since we dropped the subscript above, this generalized derivation is applicable for all regulators. It should be noted that this model has a number of assumptions and limitations; it is one of a number of models that can fit this framework.

### Simulation of the network expansion by generative network models

To explore the saturation process in a more general framework, we also simulated a generative network model to see if the same co-regulatory characteristics appeared in a computed evolutionary environment. The model was built using a probability-based move set derived from the current understanding of gene regulation network formation [1], [4], [15], [16], [17], [31], [32], [33]. The model contained two node varieties: regulators (say a transcription factor, TF) and targets (say a target gene, TG). Allowed move types included: 1) the addition of a new node (either TF or TG), 2) the duplication of a node with partial edge inheritance (an inheritance rate of 30% was used for both TFs and TGs), 3) the transformation of a TF into a TG (identified as TF-TG), which becomes regulated by another TF but still maintains all current regulatory interactions, 4) the addition or deletion of an edge between a TF and a TG, and 5) the deletion of a node (TF or TG). The model began with one TF and one TG. For each of a total of 10,000 iterations, a move was chosen on the basis of a random probability. If the move involved an action on an existing TF or TG (which included all moves except the addition of a new node), one was chosen at random from the available nodes in the network. The resulting generative network model used in this analysis contained 160 TFs and 2073 TGs. The co-regulatory network derived from this model (figure 3h) showed a similar trend to that in the model organisms. At a certain point, the number of regulatory partners began to level off even as the number of regulated targets increased, leading to the characteristic saturation curve. This indicates that the saturation curve seen in these co-regulatory networks could be a product of evolutionary development, during which regulators gain and lose interactions with targets over time.

### Controls to test robustness to methodology and incompleteness of data

One of the issues with studies dealing with the regulatory data is incompleteness. Currently, the data for many species, especially rat and mouse, is far from being complete in two respects. It is short of regulatory nodes (there are many novel regulatory factors that are expected to be discovered) and regulatory edges (more regulatory interactions between the current set of nodes are expected). We tested the robustness of the relationships reported above to both kinds of incompleteness by taking smaller random samples from the current data and repeating the analysis. In three separate trials, 20% and 40% of the nodes were randomly removed each time from the current network, as were 20% and 40% of the edges in separate runs. We found that in almost all cases, the relationship between the number of target genes and the number of partners was retained (Figures 3 through 14 in text S1). Slight deviations were observed for rat (See legend to Figure S3 in text S1). We believe that this slight disagreement is due to the fact that the information for this species is already very scarce and further removal of portions of the data makes it even scantier and thus disturbs the relationship. This is corroborated by the fact that the exponential saturation relationship as observed for the full dataset is observed for 80% of the data. It is, however, lost when only 60% of the data is retained. We also used another strategy to select statistically significant edges: we used z-score which for each pair was calculated as z = (x−μ)/σ where μ is the mean of the number of partners jointly co-regulated by the pair in 1,000 simulation of randomized networks of the same degree connectivity and σ is the standard deviation of this number. In another run, we used all the edges in the co-regulation network (no edges were removed). In both these cases, we obtained the results as above (Figures S15 and S16 in text S1). The above analysis shows that our results are more or less robust to the current incompleteness of the regulatory data.

## Discussion

A partnership network describes the associations made between two regulators that co-regulate at least one common target gene. In this study, we have revealed the topological properties of two kinds of biological partnership networks (co-transcription and co-phosphorylation) generated from the regulation network across five different species spanning a large evolutionary period. With regards to the relationship between regulatory and co-regulatory interactions, we observe differences between *E. coli* and other higher organisms. While *E. coli* shows a linear increase in co-regulatory partners as the number of target genes increases, other organisms show an exponential saturation relationship between the two quantities. We demonstrate that this apparent dissimilarity is also present in another bacterium, *B. subtilis*, and occurs because the saturation part of the curve is not reached only achieving the initial part which is linear. We believe that this is due to the differences between the architecture of the transcription programs: in bacteria, many genes are regulated by the same set of regulatory elements due to the presence of operons and this reduces the number of distinct ‘genes’ available. We have also presented a very simple model that describes the growth of these networks and explains the observed patterns.

The relationship present in the co-regulatory networks is also observed in social networks, highlighting the similarities between the architecture of social and regulatory networks. Interestingly, the above findings are more or less consistent across all five species in spite of large evolutionary distances and difference in the size/complexity of the regulatory networks. This suggests that the above properties are inherent in regulatory and co-regulatory networks of all living species. To show that our results are robust to the incompleteness of available data, we have carried out the analysis presented in this study on smaller subsamples, leading to similar observations. This demonstrates that the conclusions drawn here are unlikely to change when more data becomes available or when different values of the parameters are used.

The analysis presented in this study can be pursued further in various directions in future work. First, in addition to analyzing the co-regulatory networks using a static perspective for the five species as done here, it would be of great interest to perform the same analysis in a dynamic framework, e.g. under different conditions and stages of the cell cycle, similar to previous works that have revealed some interesting properties of the dynamic regulatory network of yeast [11]. Second, it would also be interesting to extend the analysis to add RNA interference (RNAi) where microRNAs (miRNA) at specific DNA regions to control the amount of proteins produced in the cell which would involve two types of nodes (microRNA and the proteins). There are also a number of other directions that could be pursued. We have started with the preliminary work on some of these that are sufficiently straight-forward. In particular, we performed a similar analysis as above at the target level, i.e. we created a ‘co-regulated’ network by inferring an edge between two targets if they have the same regulator. We found that there is no clear and consistent relationship between the number of partners and the number of regulators; the relationship between the two is rather noisy (Figure S17 in text S1). We also carried out an examination of the correlation between co-regulatory edges and protein-protein interactions (PPI). However, we found that there is no enrichment of co-regulation edges in the PPI network (Table S1 in text S1). Nevertheless, we believe that it might be worthwhile to pursue these directions more closely in the future when more data becomes available.

In summary, we have carried out an analysis of the co-regulatory associations made between regulators across five different species in order to analyze the organization and growth of co-regulation networks. The results presented here define the basic elements of the co-regulatory networks and given the fast computations of the quantities presented herein, we hope that the framework presented here aids in the directed investigation of the co-regulatory network in the future in order to gain deeper insight.

## Materials and Methods

### Dataset

We chose five species for the analysis: *E. coli*, yeast, mouse, rat and human. These specific species were chosen for two reasons. One, these species are evolutionarily diverse, which lends more confidence to an observation if it is true for all these species. Two, the data for these species is most plentifully available. Transcription regulatory data for *E. coli* was obtained from regulonDB version 6.2 [34]. For yeast, it was the same as used in previous similar studies [17], [18]. This data was collected from the results of genetic and biochemical experiments [2], [10], [35], [36], [37]. For rat, mouse, and human, regulatory interactions were obtained from the TRED database (as of June 2008) [38]. Human TF list in various annotations is available at http://wiki.gersteinlab.org/pubinfo/Human_TF_List. Phosphorylation data for yeast was obtained from a large scale proteome chip experiment [39]. Human modification network was obtained from HPRD that contained more than 30 kinds of post-translational modifications such as acetylation, alkylation,, carboxylation, demethylation, glycation, hydroxylation and nitration [40]. The sizes of the networks are provided in Table 1.

As for the social networks, we analyzed two types: blog and email. We obtained a network of blogs written over the period of two months preceding the U.S. Presidential Election of 2004 [41] where bloggers hyperlinked their blogs to others. This data was comprised of 1225 blogs and 19090 hyperlinks between them. The email network was obtained by analyzing the email communication within a medium sized university between 1669 users of various designations [42].

### Network transformation

We built the co-regulatory network from the regulatory network in the following way. First, an edge was placed between two regulators if they regulated the same target gene. Then we generated 1,000 random networks of the same degree distribution as the original regulatory network. In these null-networks, all proteins had exactly the same connectivity as in the original one, whereas the choice of their interaction partners was totally random, thus maintaining the in- and out-degree of each node. For every pair of regulators, we calculated the ratio of the number of target genes regulated in the real network and the average number of target genes regulated in random networks. To keep only those co-regulatory associations that are more frequent than random ones, edges with a ratio >1 were retained. As used in previous studies, this strategy removes those edges that are less probable than random [17], [18].

## Supporting Information

Text S1 Supplementary text and figures
(0.60 MB DOC)
