# Supplementary material for: Analysis of Combinatorial Regulation: Scaling of Partnerships between Regulators with the Number of Governed Targets
Source: PLoS Comput Biol. 2010 May 27;6(5):e1000755. doi: 10.1371/journal.pcbi.1000755 (PMC2877725; doi:10.1371/journal.pcbi.1000755)
Supplement: Text S1 — Supplementary text and figures (0.60 MB DOC) [file pcbi.1000755.s001.doc]

**Supplementary Materials**

**Relation of co-regulation edges to protein-protein interaction edges**

We obtained the PPI data for *E. coli* from EcoCyc and from BioGrid for the other four species. The table below provides the size of the dataset (number of edges in PPI network, 2^nd^ column) along with the size of the co-regulation dataset (number of co-regulation edges, 3^rd^ column). We find that there is no enrichment of co-regulation edges in the PPI network as shown by a very small number of overlapping edges between the two networks. None of the p-values are significant (above 0.8). Thus we hypothesize that while it is more probable for two proteins that are co-regulated by a common regulator to physically interact (as suggested by previous studies, Snel et al (2004) *Nucleic Acids Res* 32: 4725-4731), no such correlation exists at the level of regulators i.e. it is not more probable than random for two co-regulators to be involved in a PPI.

| Species | # of PPI edges | # of co-regulation edges | # of overlapping edges |
| --- | --- | --- | --- |
| *E. coli* | 74,776 | 550 | 20 |
| Yeast | 154,145 | 3000 | 92 |
| Rat | 621 | 708 | 2 |
| Mouse | 2,021 | 1,290 | 11 |
| Human | 42,219 | 2,498 | 126 |

Supplementary Table 1.


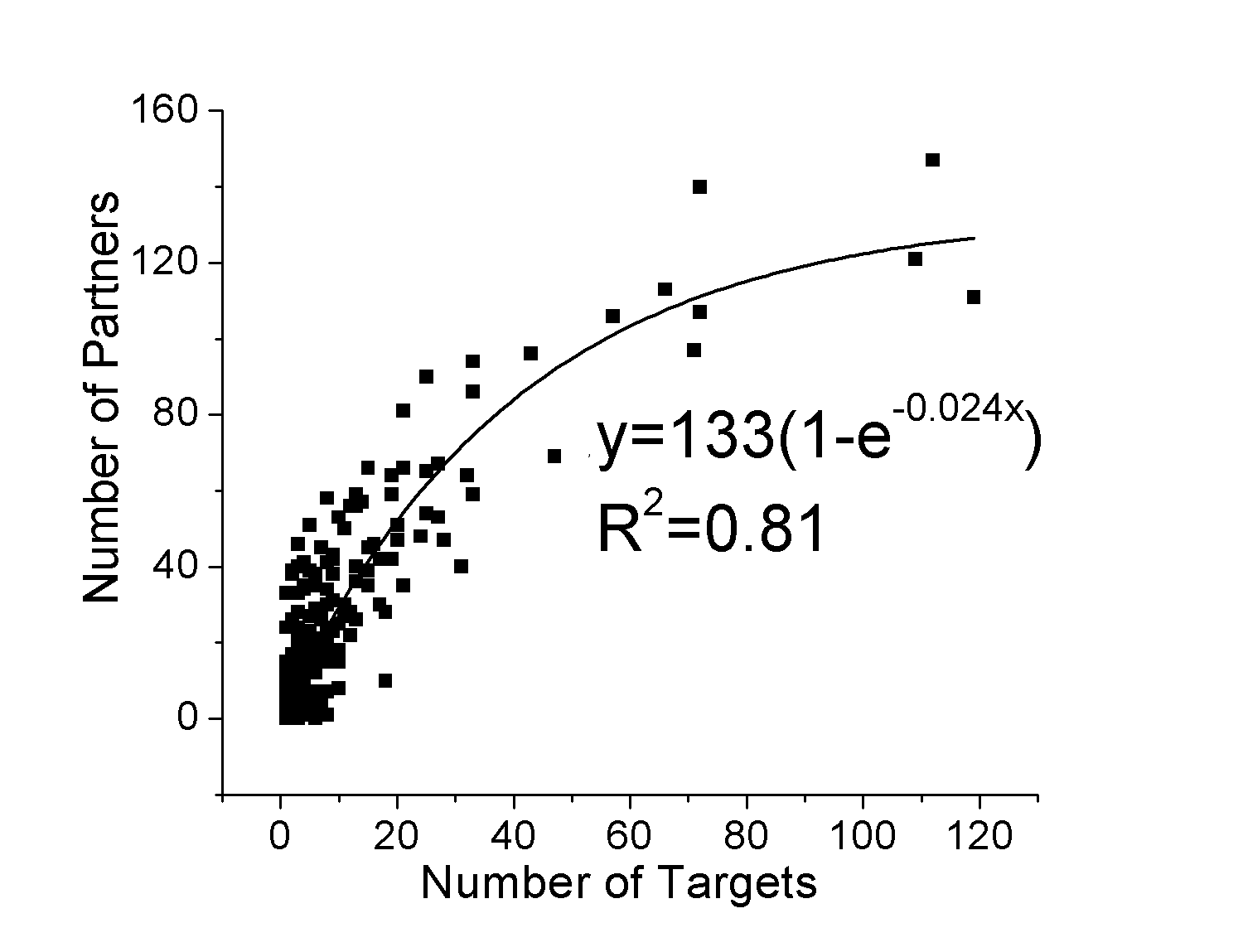


Figure 1: The number of partners vs. the number of target genes for the modification network of human. The best fit line and corresponding R^2^ value is indicated.


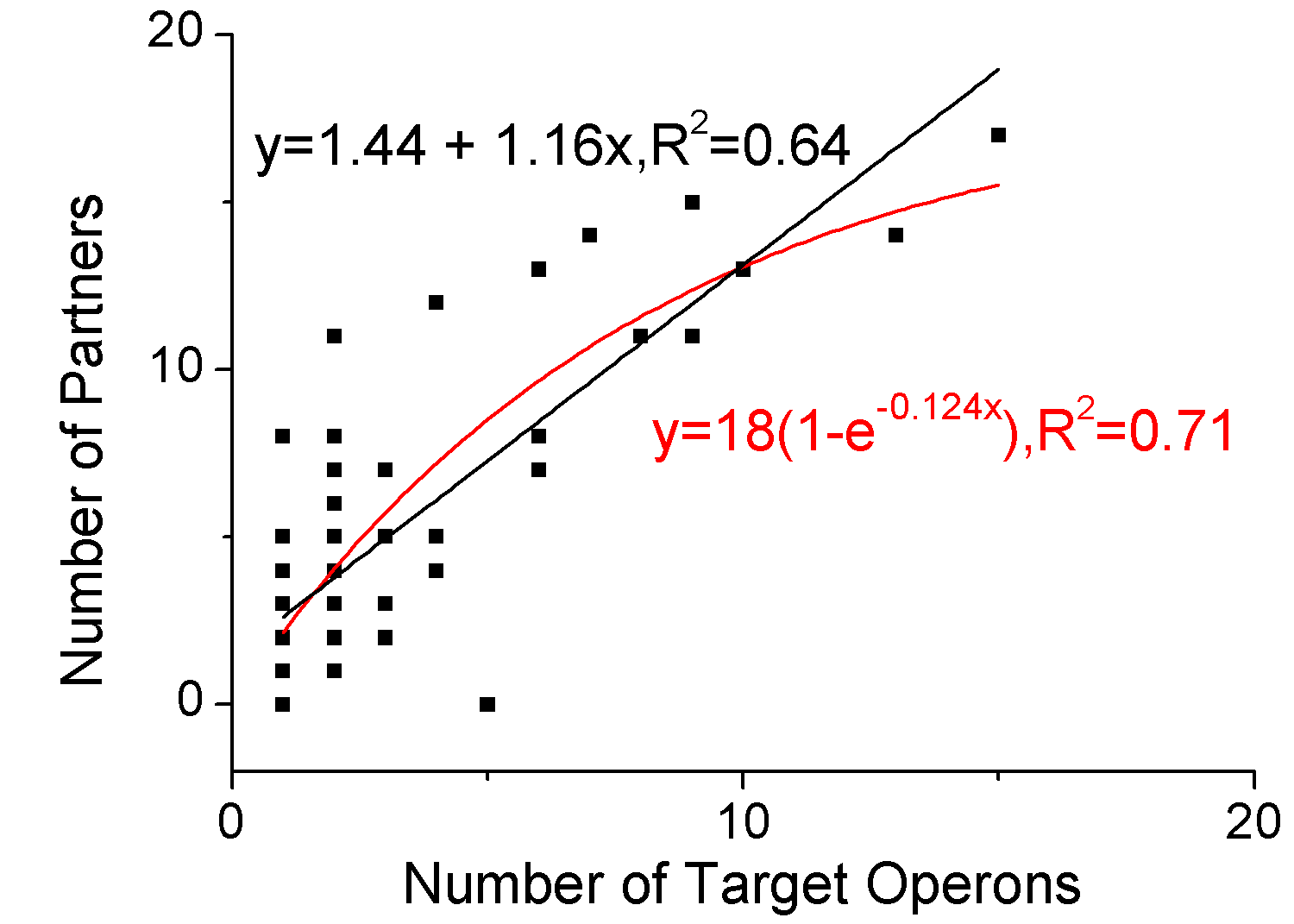


Figure 2. The number of partners vs. the number of target operons for *E. coli* when the outliers are removed. The exponential saturation curve (in red) shows a slightly better fit than the linear curve (in black) for both species.


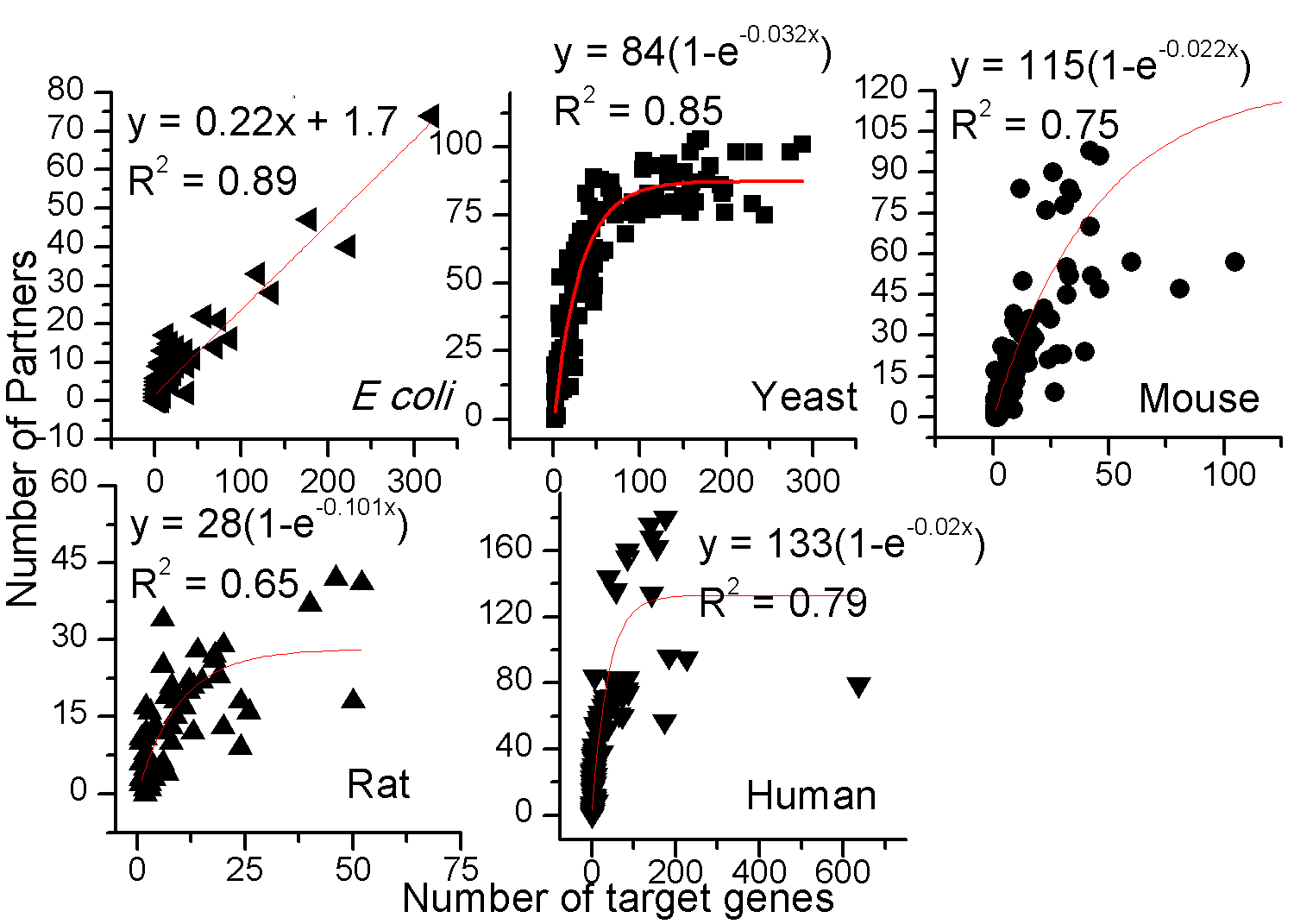


Figure 3. Number of partners vs number of target genes for each regulator in transcription regulatory network in the five species when 20% of the nodes are removed randomly (Trial 1). The best fit line is indicated for each sub-graph with the corresponding R^2^ values. In some cases, a better fitting straight line is shown with the R^2^ value.


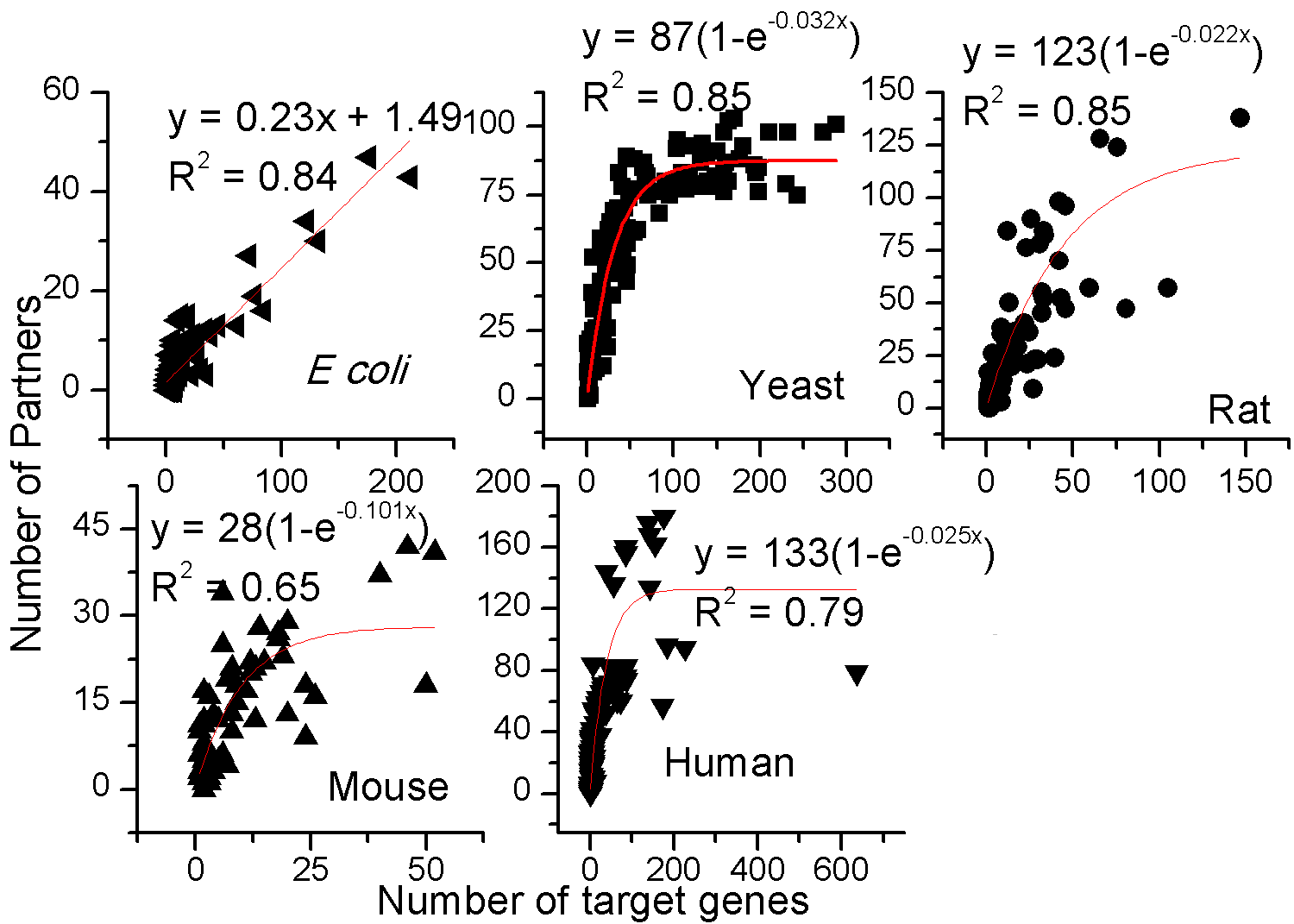


Figure 4. Number of partners vs number of target genes for each TF in the five species when 20% of the nodes are removed randomly (Trial 2).


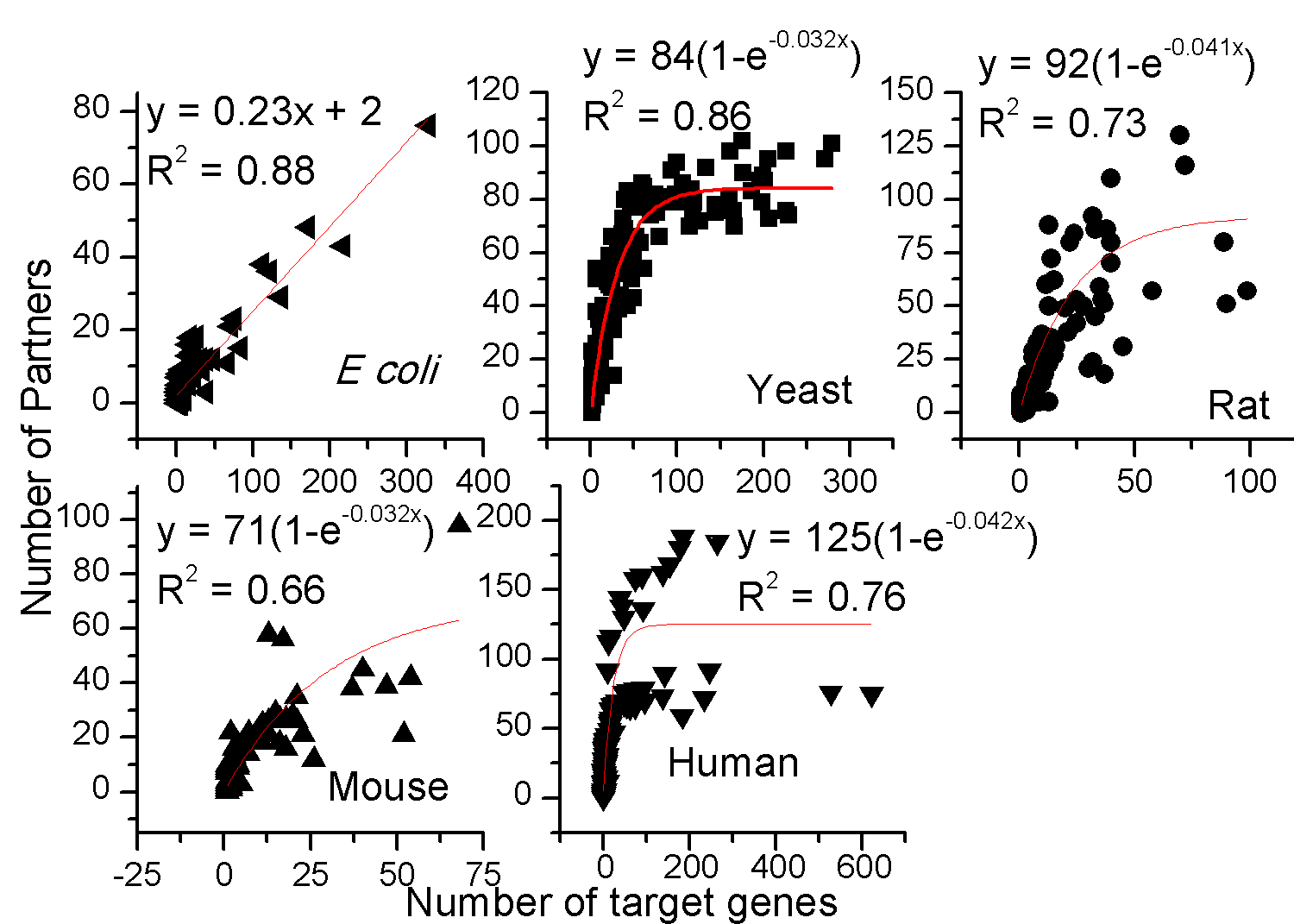


Figure 5. Number of partners vs number of target genes for each TF in the five species when 20% of the nodes are removed randomly (Trial 3).


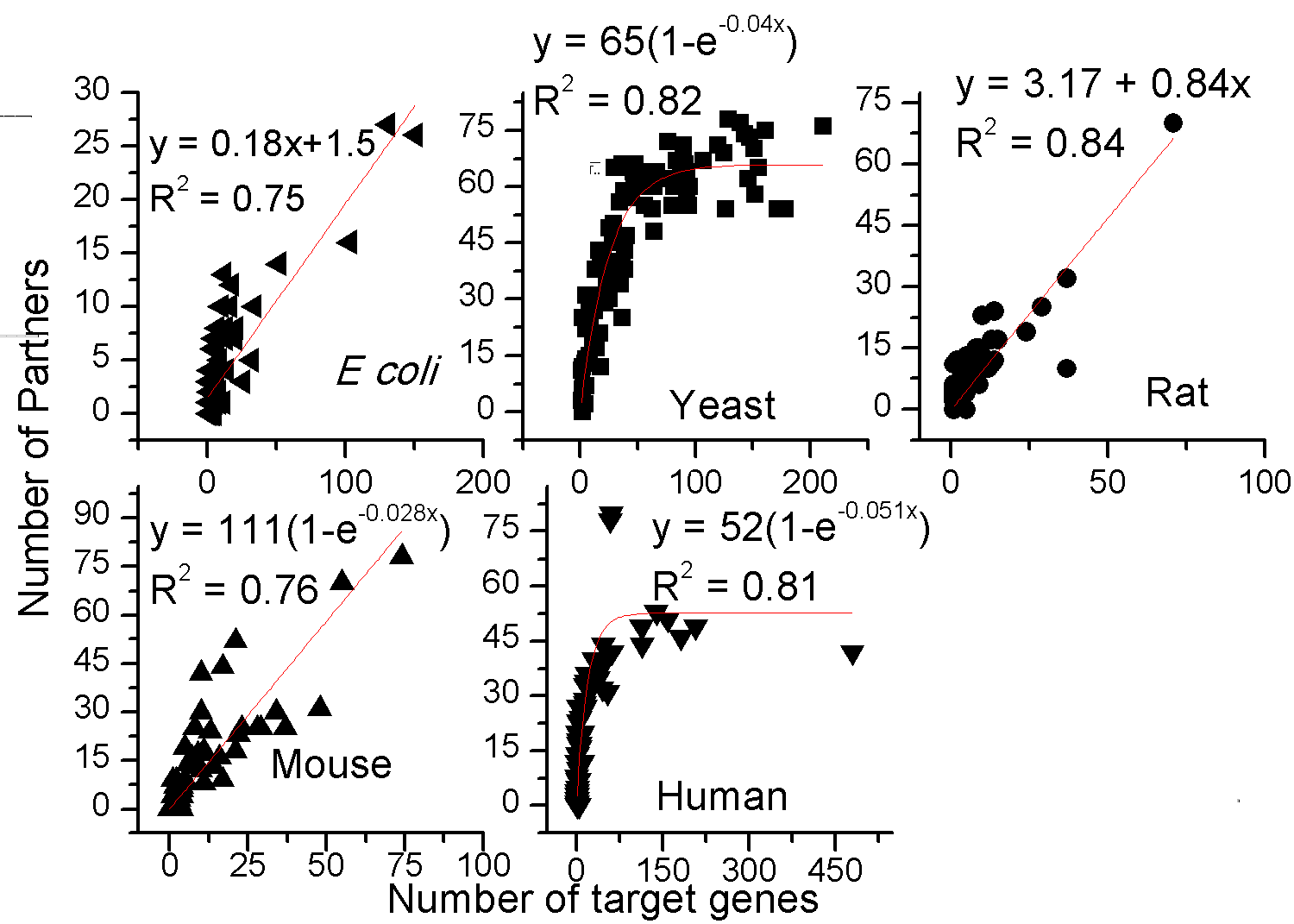


Figure 6. Number of partners vs number of target genes for each TF in the five species when 40% of the nodes are removed randomly (Trial 1).


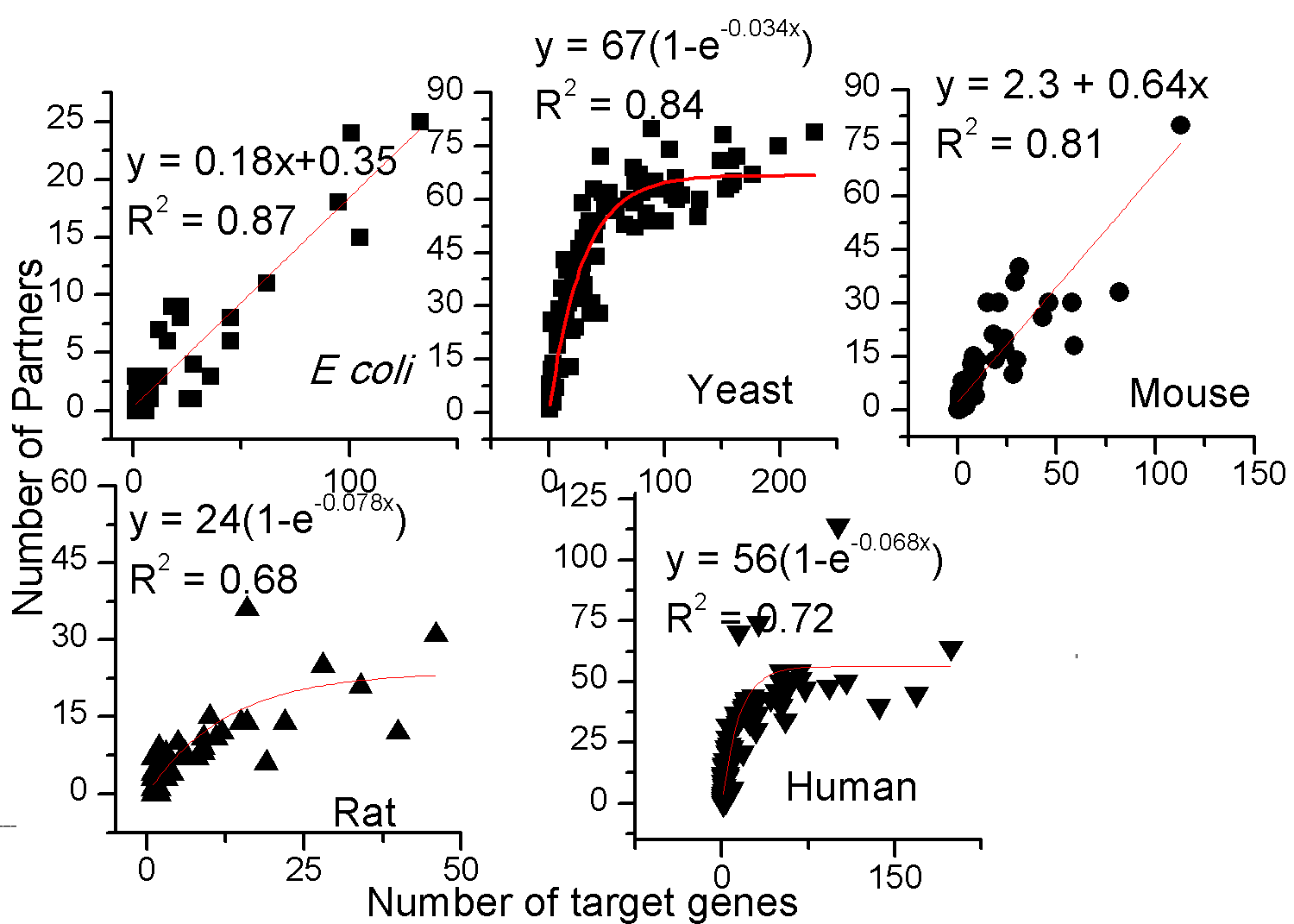


Figure 7. Number of partners vs number of target genes for each TF in the five species when 40% of the nodes are removed randomly (Trial 2).
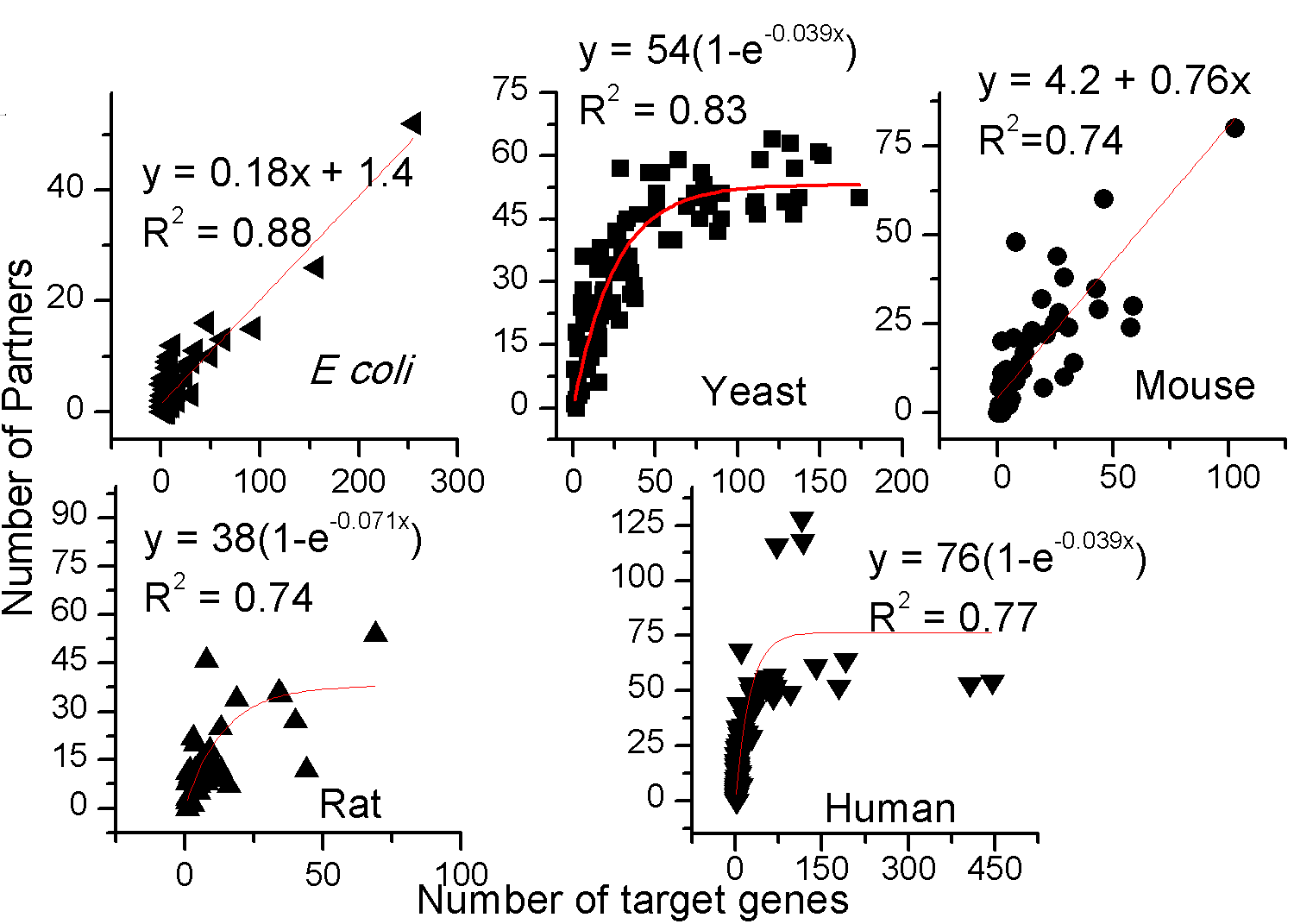


Figure 8. Number of partners vs number of target genes for each TF in the five species when 40% of the nodes are removed randomly (Trial 3).


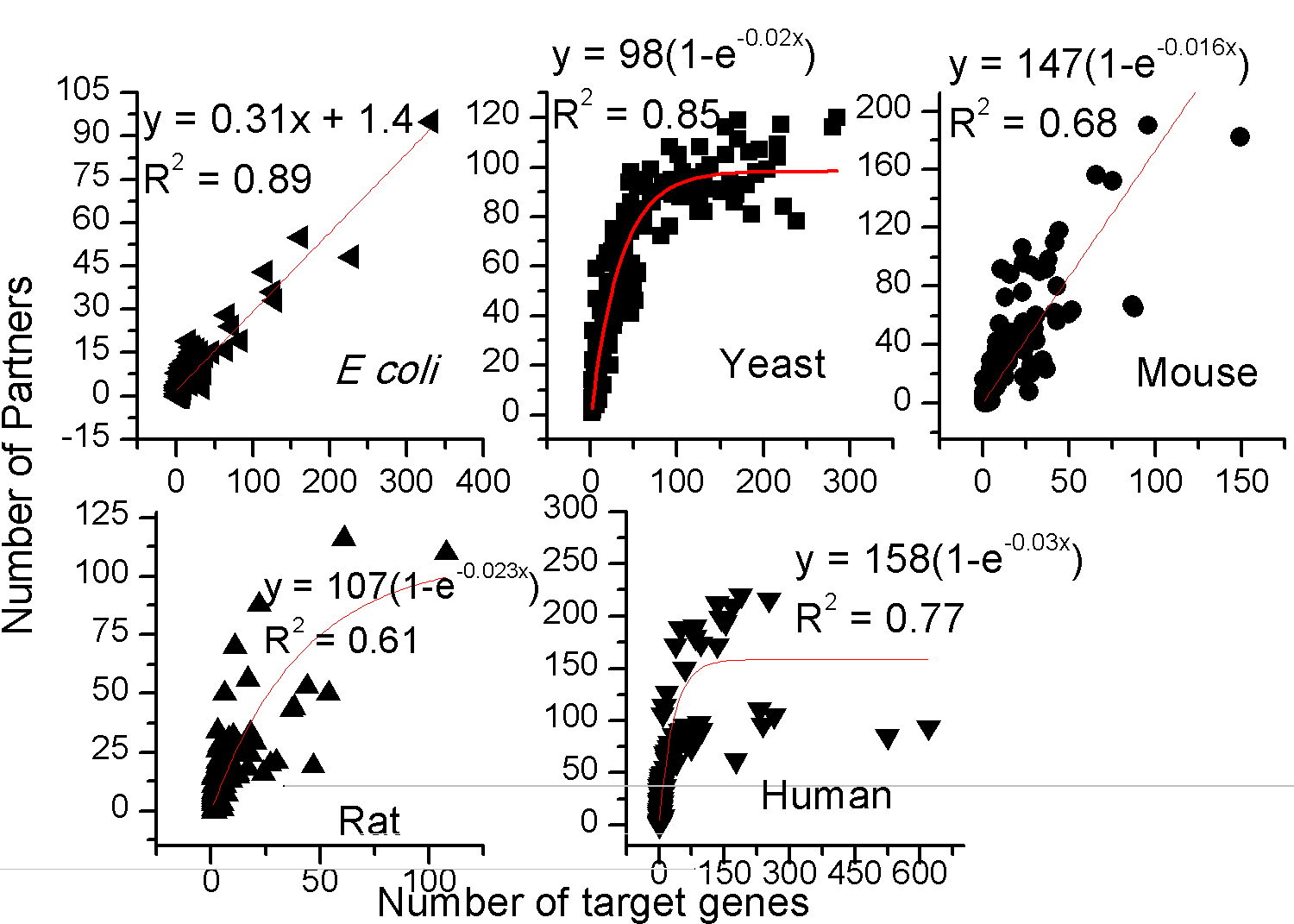


Figure 9. Number of partners vs number of target genes for each TF in the five species when 20% of the edges are removed randomly (Trial 1).
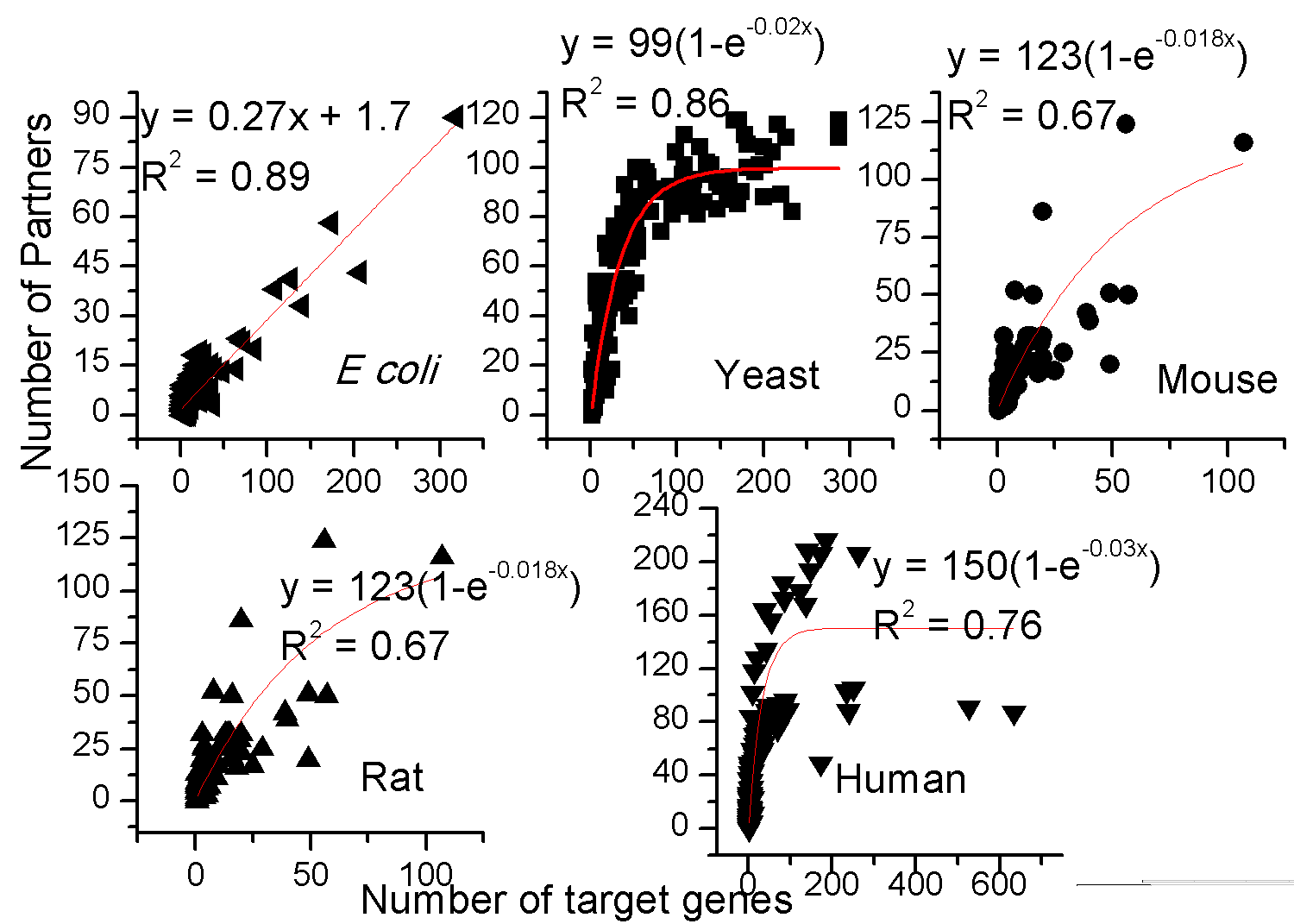


Figure 10. Number of partners vs number of target genes for each TF in the five species when 20% of the edges are removed randomly (Trial 2).
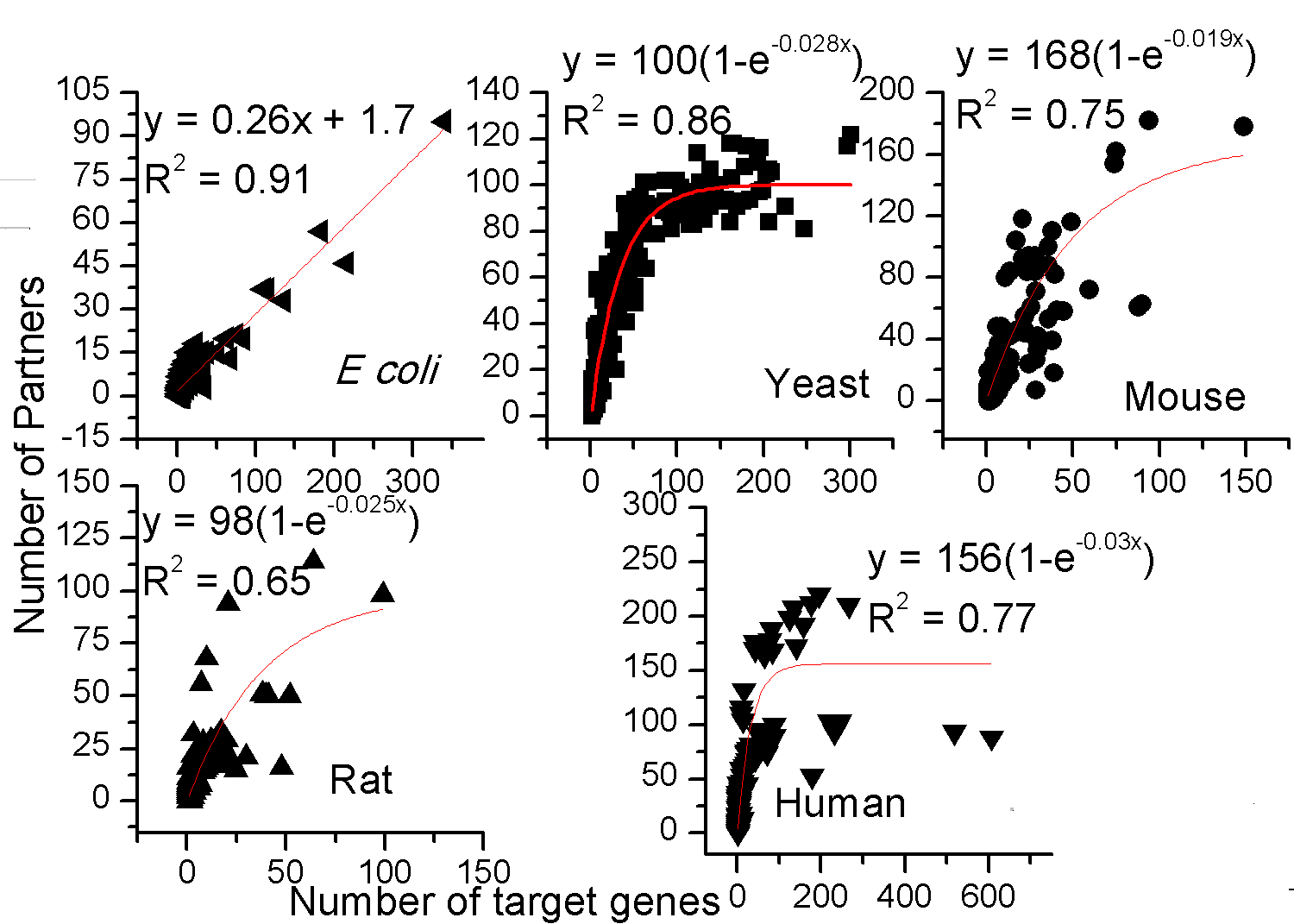


Figure 11. Number of partners vs number of target genes for each TF in the five species when 20% of the edges are removed randomly (Trial 3).
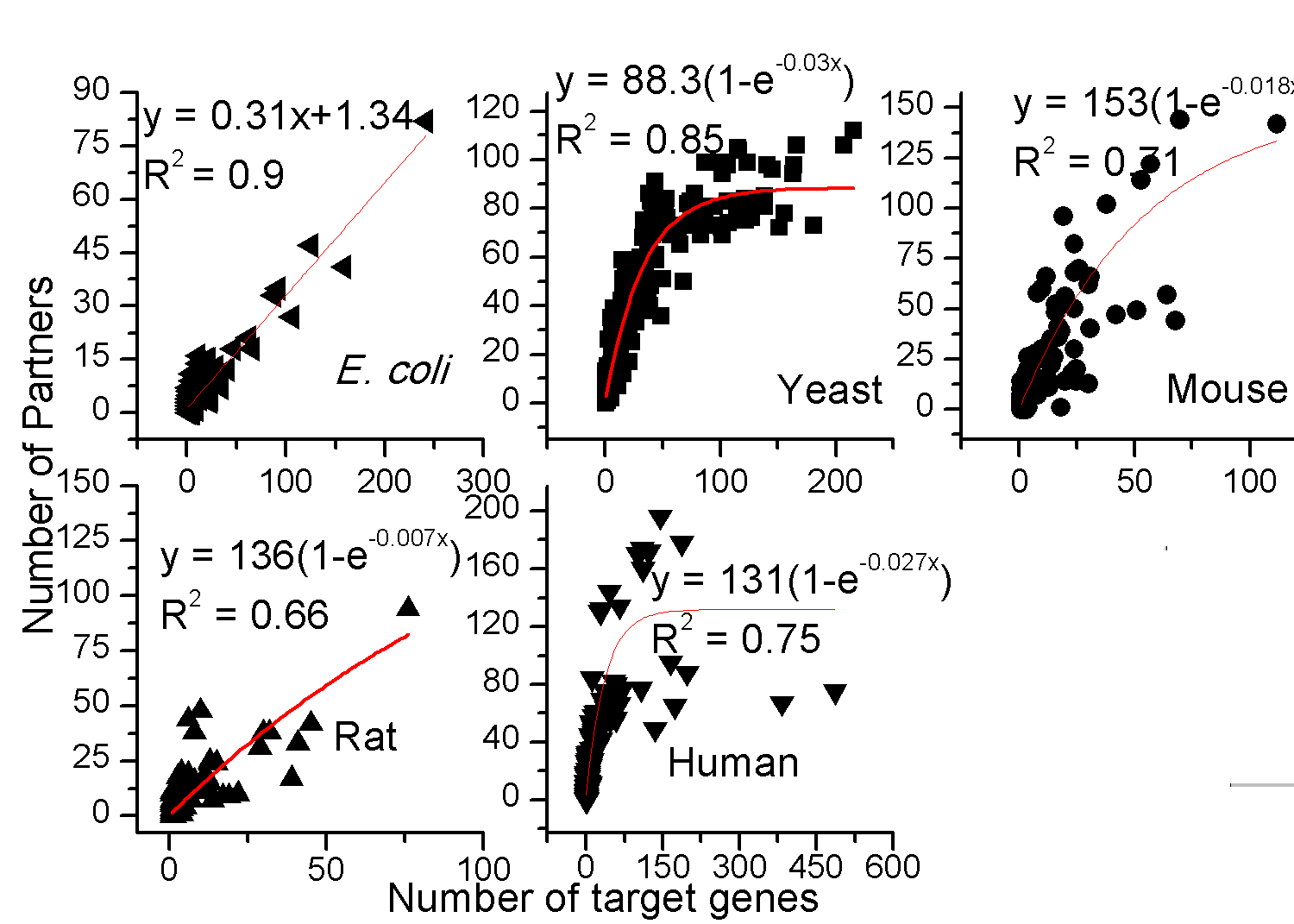


Figure 12. Number of partners vs number of target genes for each TF in the five species when 40% of the edges are removed randomly (Trial 1).
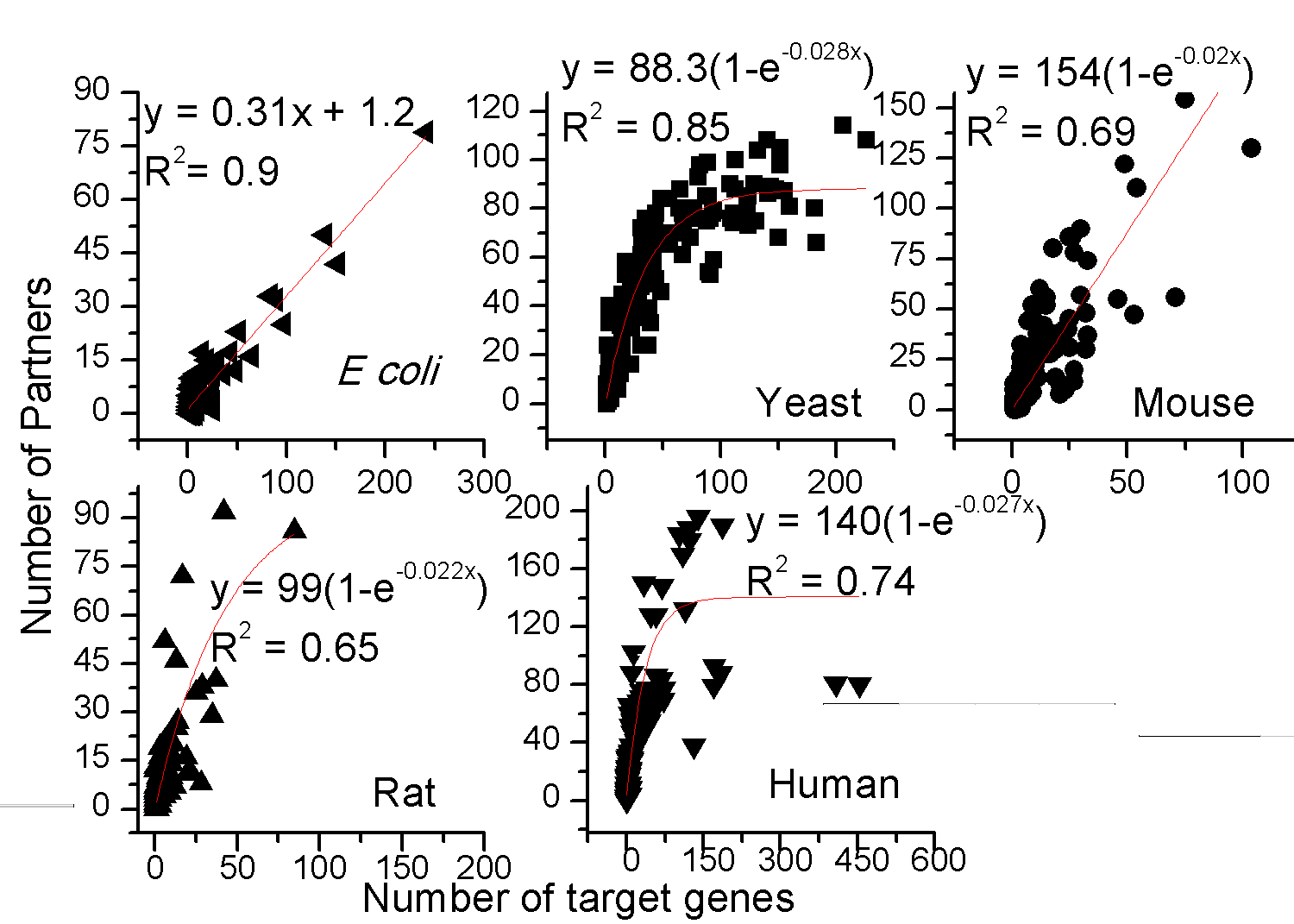


Figure 13. Number of partners vs number of target genes for each TF in the five species when 40% of the edges are removed randomly (Trial 2).
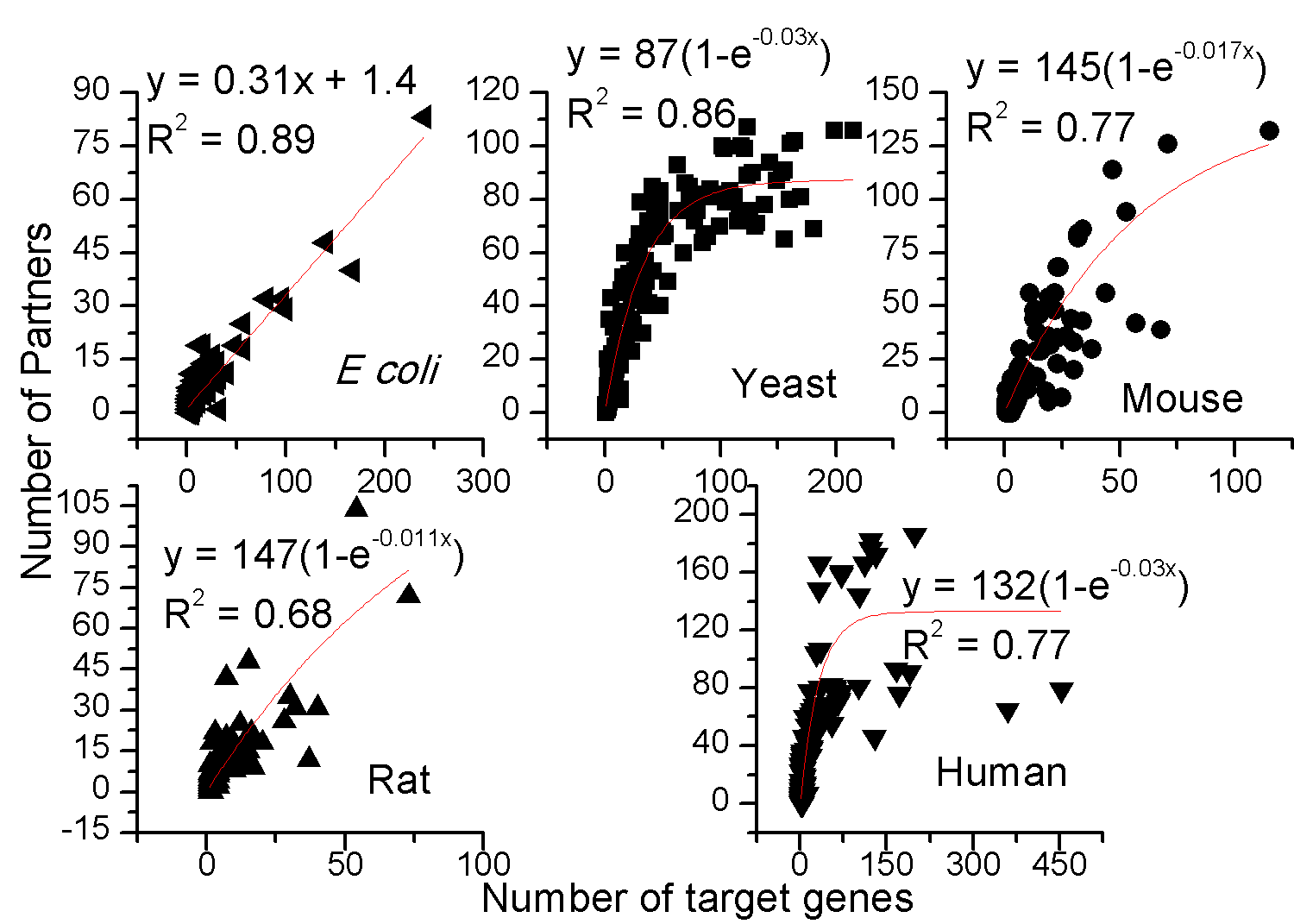


Figure 14. Number of partners vs number of target genes for each TF in the five species when 40% of the edges are removed randomly (Trial 3).


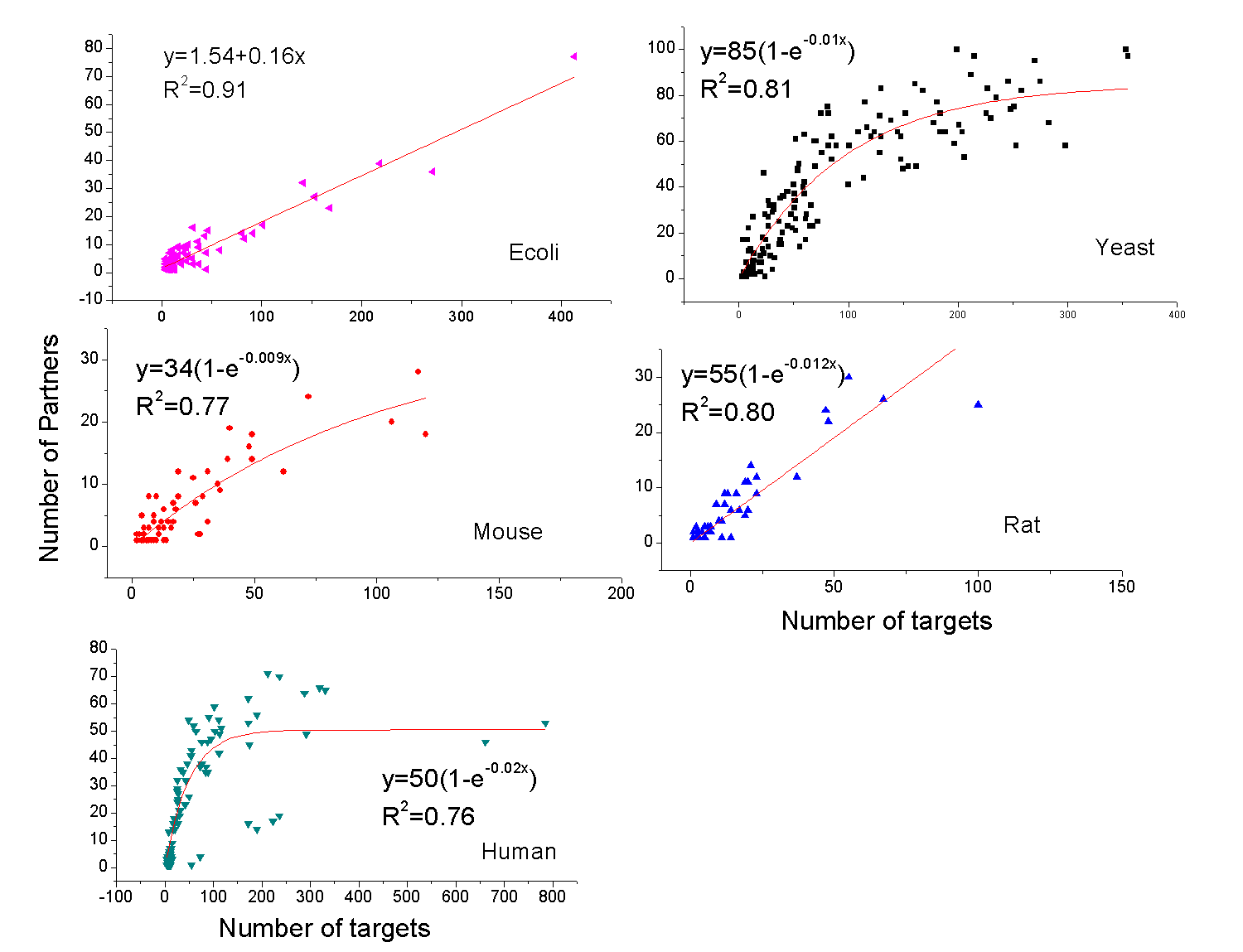


Figure 15. The number of partners vs. the number of target genes for each regulator in the transcription network of five species when edges are considered (no non-significant edges are removed). The best fit line and corresponding R^2^ value is indicated for each sub-graph. The z-score for each pair is calculated as z=(x-μ)/σ where μ is the mean of the number of partners jointly co-regulated by the pair in 10,000 simulation of randomized networks of the same degree connectivity and σ is the standard deviation of this number.


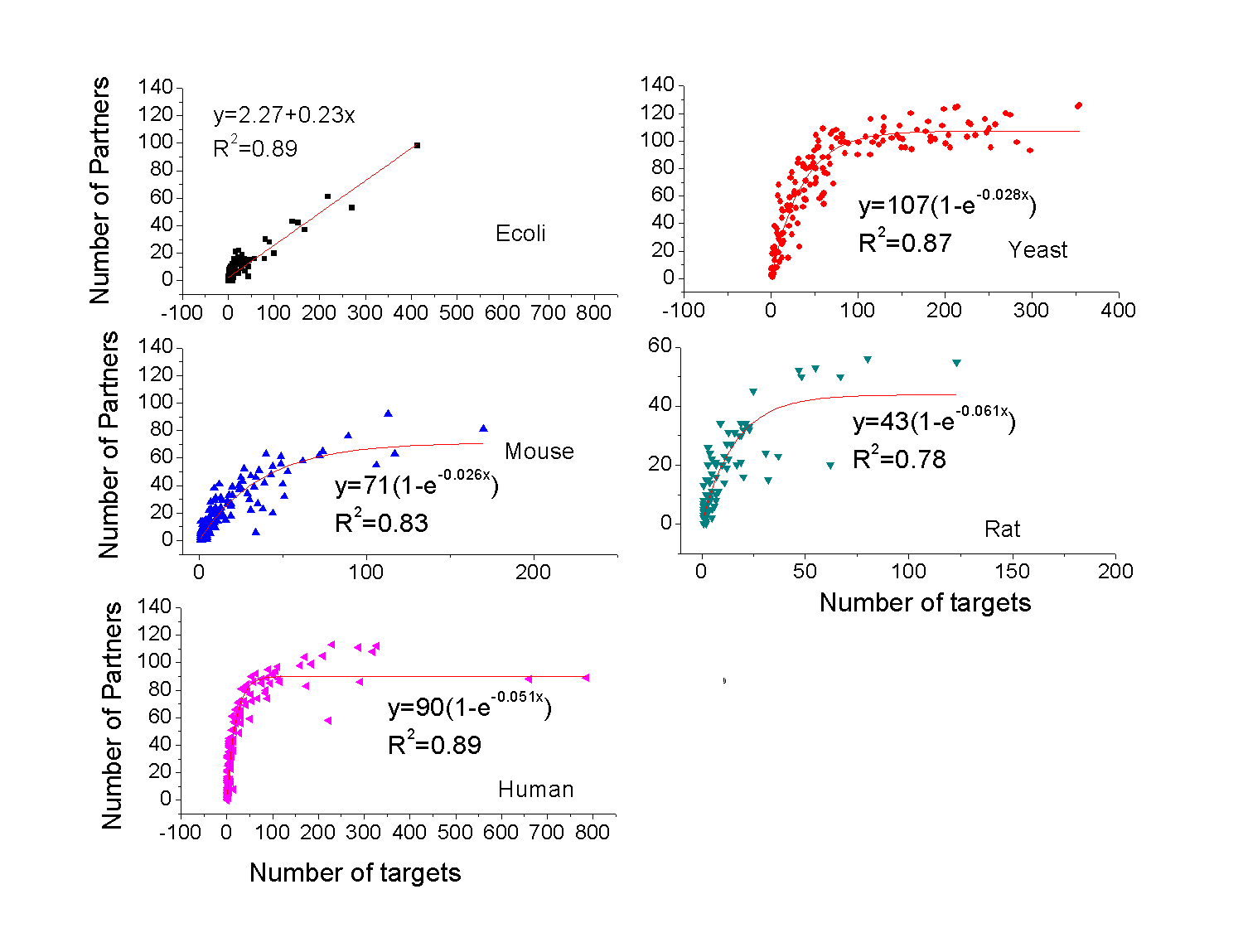


Figure 16. The number of partners vs. the number of target genes for each regulator in the transcription network of five species when all the edges are considered (no non-significant edges are removed). The best fit line and corresponding R^2^ value is indicated for each sub-graph.


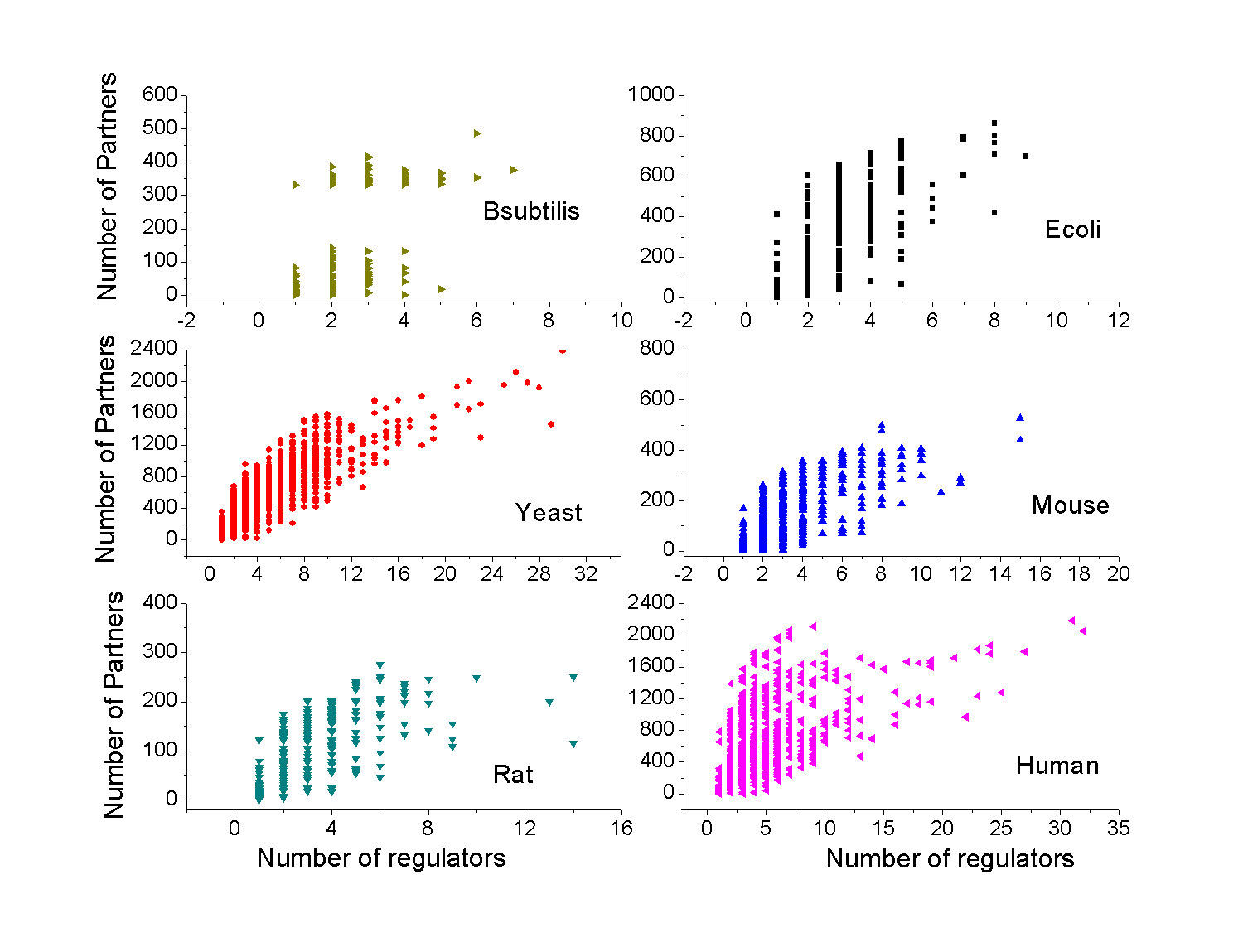


Figure 17. The number of partners vs. the number of regulators for each target gene in the transcription network of the six species.
